# Recycling and Reutilization of Waste Carbon Fiber Reinforced Plastics: Current Status and Prospects

**DOI:** 10.3390/polym15173508

**Published:** 2023-08-23

**Authors:** Pi-Yu Chen, Ran Feng, Ying Xu, Ji-Hua Zhu

**Affiliations:** 1 School of Civil and Environmental Engineering, Harbin Institute of Technology, Shenzhen 518055, China; 19b954012@stu.hit.edu.cn (P.-Y.C.); cexyx@hotmail.com (Y.X.); 2 Guangdong Provincial Key Laboratory of Durability for Marine Civil Engineering, College of Civil and Transportation Engineering, Shenzhen University, Shenzhen 518060, China

**Keywords:** carbon fiber reinforced plastics (CFRP) waste, recycled carbon fiber reinforced cementitious (rCFRC) composites, recycled carbon fiber reinforced plastics (rCFRP), recycling methods, reuse of recycled carbon fibers (rCFs)

## Abstract

The extensive use of carbon fiber-reinforced plastics (CFRP) in aerospace, civil engineering, and other fields has resulted in a significant amount of waste, leading to serious environmental issues. Finding appropriate methods for recycling CFRP waste and effectively reusing recycled carbon fibers (rCFs) has become a challenging task. This paper presents an overview of the current status of CFRP waste and provides a systematic review and analysis of recycling technologies. In addition to discussing mechanical recycling, thermal decomposition, and chemical solvent degradation methods, the organic alkali/organic solvent method for recycling resins is also elucidated. By introducing the recycling conditions and outcomes of the organic alkali/organic solvent method, the study highlights its significance as a reference for carbon fiber recycling. Furthermore, the paper reviews the current state of rCFs utilization based on its application domains, focusing on research advancements in fiber composites and cementitious composites. Based on these findings, the paper summarizes the existing research limitations and identifies specific areas that require further attention in recycling techniques and rCFs utilization. Lastly, this review provides a prospect on the future of recycling and reusing CFRP waste.

## 1. Introduction

**Figure 1:**
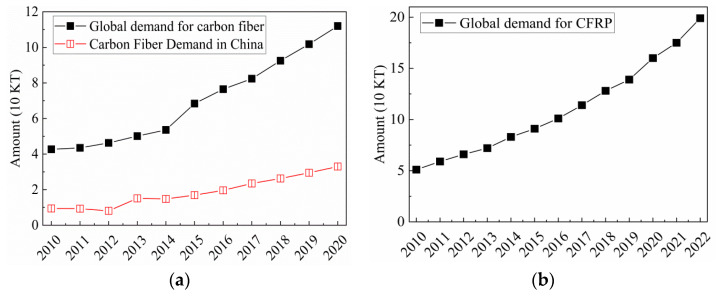
(**a**) Demand for carbon fiber [3]; (**b**) Demand for CFRP [4].

Carbon fiber reinforced plastics (CFRP) is a composite engineering material produced through carbon fibers as reinforcements and resin as the matrix. CFRP exhibits numerous advantages, including high strength, high stiffness, low density, and corrosion resistance [1,2]. Due to these properties, CFRP finds extensive applications in industries such as aerospace, civil engineering, wind power generation, sports and leisure, rail transportation, and pressure vessels. Since the beginning of the 21st century, there has been a rapid increase in global demand for carbon fibers. According to statistics, the demand has grown from 43,500 tons in 2012 to 82,400 tons in 2017 and reached 112,000 tons in 2020 [3]. In China, the demand for carbon fibers has maintained an annual compound growth rate of over 15% in recent years, reaching 33,000 tons in 2020. As a product of carbon fibers, the global demand for CFRP reach 199,000 tons in 2022 [4]. The demand for carbon fibers and CFRP is illustrated in Figure 1.

However, the extensive consumption of CFRP and the expectation of continuous consumption growth have resulted in an enormous accumulation of CFRP waste. Pimenta and Pinho [5] revealed that by 2020, retired CFRP products would reach approximately 26,000 tons, with manufacturing process waste amounting to 36,000 tons. Carberry [6] predicted a surge in the retirement of commercial carbon fiber aircraft by 2025, with an estimated quantity of 8500 aircraft, providing over 12,000 tons of recyclable CFRP. By 2050, the global CFRP waste is projected to reach a staggering 500,000 tons [7]. The resin in CFRP forms a three-dimensional cross-linked network structure, rendering it insoluble and non-melting, while carbon fiber exhibits inert characteristics. Consequently, CFRP is highly resistant to natural degradation. Traditional disposal methods primarily involve landfilling and incineration. However, over a hundred years, CFRP degradation in landfills would only account for 1% of its original mass, and it would take 60,000 years to degrade by 26% [5]. Incinerating one ton of CFRP would produce 2011 kg of CO_2_ [8]. Therefore, adopting the conventional strategies of landfilling and incineration not only consumes vast valuable land resources but also results in a CO_2_ production of over 200,000 tons in 2022, causing significant environmental pollution and conflicting with the long-term vision of carbon emissions neutrality. Typically, carbon fibers in waste maintain their mechanical properties to a large extent before recycling, and the use of appropriate recycling methods can achieve performance similar to virgin carbon fibers (vCFs). In 2020, the total CFRP waste reached 62,000 tons [5]. Approximately 600 kg of carbon fiber can be recovered from one ton of CFRP waste. Based on this calculation, approximately 37,200 tons of carbon fiber could be recovered in 2020, representing around 33.2% of the carbon fiber demand for that year. Therefore, CFRP waste holds tremendous potential for utilization.

Faced with the serious issue of CFRP waste and recognizing its significant potential for utilization, governments worldwide, particularly those of developed nations, have successively implemented regulations and policies to guide and encourage proper waste management by businesses. In 1999, the European Union (EU) introduced a policy [9] that unequivocally assigned responsibility to CFRP manufacturers for handling their products and strictly limited the amount of CFRP waste sent to landfills. Furthermore, tax policies were devised to incentivize businesses to engage in CFRP composite material recycling [10]. Since the beginning of the 21st century, policies about CFRP waste management have further tightened. Both the EU Management Committee and the United States Environmental Protection Agency (EPA) began prohibiting the landfill disposal of CFRP materials in 2004, while the UK initiated the imposition of a landfill tax on CFRP waste in 2014. In recent years, China has experienced a sharp increase in the manufacturing and use of carbon fiber-reinforced composite materials, leading to a pronounced issue of CFRP waste. Consequently, the country has released a plethora of targeted policies and regulations. The Ministry of Industry and Information Technology has mandated the promotion of CFRP recycling and reuse [11,12,13], while ministries such as the State Council and the National Development and Reform Commission (NDRC) have called for active endeavors in recycling and utilizing novel types of waste, including carbon fiber composites, along with the establishment of application demonstrations to advance the development of the resource recycling industry system [14,15,16].

The issue of CFRP waste has been escalating, while it also holds tremendous value. Coupled with the pressure from policies, the recycling of CFRP waste has become an urgent matter. Researchers have conducted studies focusing on the performance of carbon fiber recycling in terms of energy consumption, environmental impact, and costs. Carbon fibers are manufactured from polyacrylonitrile through a series of high-temperature production processes, with an energy consumption of approximately 183–594 MJ/kg [17,18]. By combining a life cycle assessment model with experimental results, the energy consumption in the carbon fiber recycling process has been evaluated. Mechanical recycling methods have an energy consumption of about 0.27–2.03 MJ/kg [19], thermal decomposition recycling methods have an energy consumption of about 3–30 MJ/kg [20,21,22], and chemical solvent degradation methods have an energy consumption of about 19.2–91 MJ/kg [23,24,25]. The energy consumption in the carbon fiber recycling process does not exceed 20% of the total energy required for vCFs manufacturing, making the utilization of rCFs to replace vCFs an effective way to reduce energy consumption. Furthermore, Akbar et al. [26], through a life cycle impact assessment, compared the environmental impact of the production processes between vCFs and rCFs. They found that the environmental impact of rCFs on non-renewable energy, greenhouse gases, ozone layer depletion, and aquatic acidification is only 4%, 12%, 24%, and 12% of that caused by vCFs, respectively. In comparison to vCFs, rCFs have minimal harmful effects on the environment [27]. Additionally, the production cost of carbon fibers is approximately USD 33 per kilogram [6,28], and the recycling cost is usually around 60–70% of the production cost of vCFs [5], mainly attributed to solvent, electricity, and equipment depreciation expenses. ELG Carbon Fibre Ltd. has achieved a 40% reduction in carbon fiber recycling costs compared to the production cost of vCFs [28]. When the production capacity reaches 100,000 kg per year, the carbon fiber recycling cost further decreases to only USD 15 per kilogram. The rCFs hold significant advantages over vCFs in terms of energy consumption, environmental impact, and cost. Therefore, the recycling and reuse of CFRP waste are feasible and practical solutions.

This paper begins by introducing the status of CFRP waste and relevant waste treatment policies. Subsequently, the feasibility of recycling and reusing CFRP waste is discussed. The second section categorizes the research on CFRP recycling into mechanical recycling, thermal decomposition recycling, and chemical solvent degradation. A comprehensive review and analysis of these methods are provided. Particular emphasis is placed on the atmospheric solvent method in chemical solvent degradation recycling, highlighting that mild recycling conditions represent a future development direction for recycling technologies. Moreover, advancements in resin recovery techniques have been recognized, leading to lowered recycling requirements and improved recycling efficiency. As a result, these resin recovery methods are grouped under the category of organic alkali/organic solvent methods within the framework of chemical solvent degradation recycling, offering valuable insights for researchers focusing on the recycling of CFRP. The third section outlines the current status of rCFs utilization according to their application areas. The research progress on rCFs in fiber composite and cementitious composite is outlined. Finally, this review concludes by summarizing the limitations of recycling techniques and the reuse of rCFs. It proposes specific areas of focus for future research and provides an outlook on the prospects of recycling and reusing CFRP waste.

## 2. Research Status and Analysis of Recycling of Carbon Fiber Reinforced Plastics (CFRP) Waste

Substantial utilization of carbon fiber and its composites began in the 1960s, primarily in the military sector, gradually extending to civilian applications during the 1970s. As the service life of CFRP is typically 15–20 years, the issue of CFRP waste emerged in the 1990s, prompting research into carbon fiber recycling. Researchers attempted mechanical recycling by collecting and crushing CFRP waste, using the resulting particles as fillers. This method is referred to as mechanical recycling. Subsequently, thermal decomposition recycling, represented by fluidized bed technology, emerged, achieving higher levels of carbon fiber and resin separation. With a deeper understanding of epoxy resin properties and technological advancements, high-temperature and high-pressure super/subcritical conditions, along with various chemical solvents, were applied to carbon fiber recycling, developing into the chemical solvent degradation recycling method. Today, a diverse range of recycling techniques has been developed, significantly improving recycling efficiency. These recycling methods can still be roughly categorized into three main types: mechanical recycling, thermal decomposition recycling, and chemical solvent degradation recycling, based on their characteristics. Their key technical indicators are presented in Table 1, which will be elaborated upon in this chapter.

### 2.1. Research on Mechanical Recycling Method

**Figure 2:**
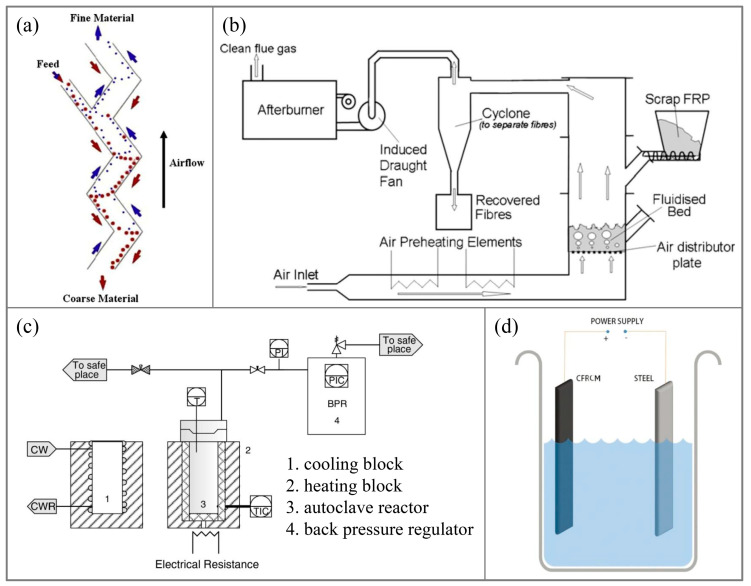
(**a**) Air classifier for mechanical recovery [30]; (**b**) Fluidized bed device [33]; (**c**) Supercritical recycling device [40]; (**d**) Electrochemical recycling device [51].

The mechanical recycling method involves initially crushing CFRP waste into fine particles containing both fibers and resin using a cutting machine. Subsequently, an air classifier is utilized to separate these components, yielding carbon fibers and resin. The purity of the rCFs depends on the effectiveness of the crushing and separation processes, which can vary significantly. Typically, CFRP is first crushed into primary products (50–100 mm) under low-speed conditions, followed by further crushing into smaller particles (below 10 mm) under high-speed conditions. These particles are then sent to the air classifier for separation, resulting in coarse material enriched with carbon fibers and powdered material enriched with resin [8,29]. Palmer et al. [30] achieved improved separation of carbon fibers and resin by progressively crushing CFRP and employing Z-type separation technology to categorize particles based on shape, weight, and other criteria, adjusting the air velocity. This approach reduces damage to the rCFs and enhances their mechanical properties, such as flexural strength. The Z-type air classifier is illustrated in Figure 2a. Mohama et al. [56] crushed two types of waste carbon fiber prepregs, partially cured and fully cured, into fine particles of 5–10 mm and enhanced polypropylene with a 3–5% content, resulting in a composite material with slightly higher tensile strength than pure polypropylene. Howarth et al. [19], through simulation, investigated the energy consumption during the mechanical recycling process and found a scale effect on energy consumption. When the CFRP recycling capacity was 10 kg/h, the energy consumption was 2.03 MJ/kg. However, when the recycling capacity increased to 150 kg/h, the energy consumption decreased to 0.27 MJ/kg.

The mechanical recycling method is characterized by its simplicity and relatively low cost. However, the separation of rCFs and resin is not thorough, resulting in low purity and short fiber length for the rCFs. Typically, these rCFs can only serve as fillers for carbon fiber composites or other products, making them of limited value. Moreover, the recycling process generates a considerable amount of resin dust, which not only pollutes the environment but also poses a significant health hazard to operators. Currently, research on the mechanical recycling method has stagnated, but it continues to find practical applications. This approach is commonly employed directly to crush CFRP, producing particles used in manufacturing resin thermal pressing plates or reinforcing cementitious composites.

### 2.2. Research on the Thermal Decomposition Method

The thermal decomposition recycling method involves subjecting waste to high-temperature conditions, causing the resin in CFRP to decompose into small molecules and gasify, thus obtaining carbon fibers. This method is capable of decomposing and separating impurities abundant in CFRP, such as different types of thermoplastic resins, paints, metals, etc. Due to its ease of large-scale application, it is currently the only CFRP recycling technology that has moved toward commercial use. The thermal decomposition recycling method mainly includes fluidized beds, gas pyrolysis (in an inert or oxygen atmosphere), and microwave pyrolysis techniques.

#### 2.2.1. Fluidized Bed Method

The fluidized bed method involves first crushing CFRP into particles and then placing them in a fluidized bed. In the flowing medium, the resin in the CFRP particles undergoes thermal decomposition. Subsequently, a fan is used to separate the carbon fibers and resin (in the form of gas or powder particles), with carbon fibers collected in a designated collector, while the resin is typically directly incinerated for disposal. Regarding the fluidized bed technique, extensive research has been conducted by the team led by S. J. Pickering from the University of Nottingham [31,32]. Yip et al. [57] subjected CFRP particles to a fluidized bed at 450 °C with a flow velocity of 1 m/s for recycling, resulting in carbon fibers with lengths shorter than 10 mm. This study found that the longer the vCFs dimensions in the waste, the higher the length of damage during the recycling process. Compared to the vCFs, the rCFs exhibited nearly unchanged Young’s modulus, but their tensile strength retained only approximately 75%. Research by Jing et al. [33] similarly indicated that carbon fibers obtained through fluidized bed recycling had minimal length retention and suffered a severe reduction in tensile strength, retaining only 50–75% of the vCFs’ tensile strength. The fluidized bed setup is illustrated in Figure 2b. Additionally, XPS testing results showed that the recycling process hardly introduced any oxygen to the carbon fiber surface, and the oxygen-to-carbon ratio on the carbon fiber surface remained largely unchanged, resulting in the interface shear strength of the rCFs being similar to that of the vCFs.

The fluidized bed method typically employs a semi-enclosed apparatus with temperatures below 500 °C, making it a relatively low-temperature thermal decomposition recycling technology in its early stages of development. The semi-enclosed structure of the fluidized bed results in a low energy utilization efficiency, leading to high energy consumption. Moreover, the pyrolysis gases are directly discharged into the environment, making collection and treatment challenging. Additionally, to maintain the fluidity and heating efficiency of the carbon fibers, their length needs to be kept below 10 mm. As a result, the resin degreasing rate for rCFs remains relatively low, and the retention of tensile strength is limited. Consequently, with the availability of closed containers and more mature heating technologies, the fluidized bed method has gradually been phased out.

#### 2.2.2. Gas Pyrolysis Method

**Figure 3:**
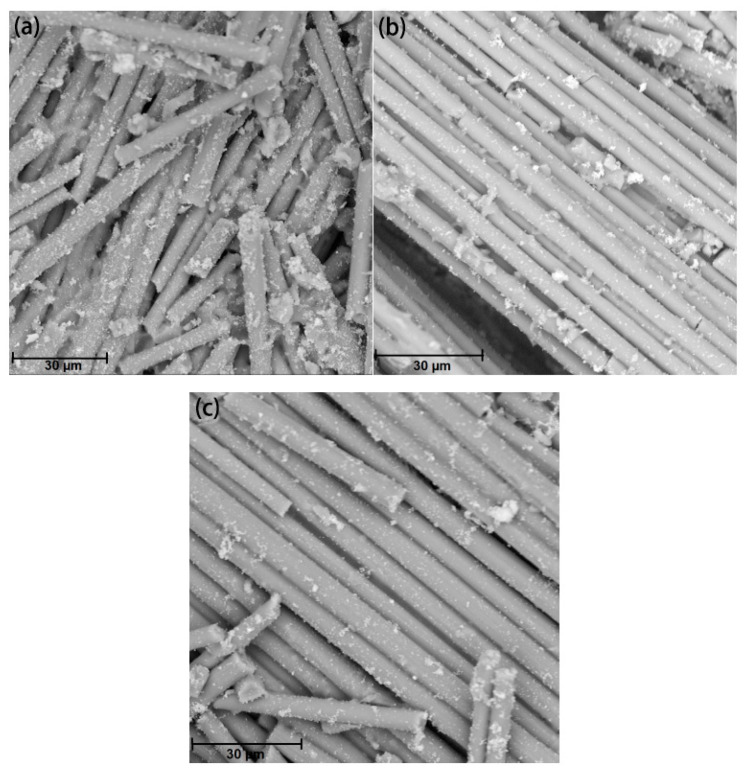
Recycled carbon fibers [35]: (**a**) 480 °C; (**b**) 520 °C; (**c**) 560 °C.

Gas pyrolysis is commonly employed to recover carbon fibers from CFRP. In this process, CFRP is placed inside a sealed container and subjected to an inert gas, such as nitrogen, at temperatures ranging from 500 to 800 °C. This causes the resin to decompose and gasify, yielding the rCFs. Schwarz et al. [34] performed thermal pyrolysis of prepreg materials in a nitrogen atmosphere at temperatures between 480 and 560 °C, resulting in the resin being thermally decomposed into gas after 1–2 h. The obtained rCFs were fragmented and displayed a surface heavily populated with resin particles. Even at 560 °C, the resin content in the rCFs remained as high as 22.6%. Similarly, Giorgini et al. [35] conducted pyrolysis of prepreg and CFRP waste in a nitrogen atmosphere at temperatures ranging from 450 to 600 °C, with a holding time of 150 min. Scanning Electron Microscopy (SEM) revealed a small amount of heterogeneous resin and carbon deposits remaining on the surface of the rCFs, as shown in Figure 3. Subsequently, both types of rCFs were placed in oxygen atmospheres at 500 °C and 600 °C, respectively, to eliminate any residual surface resin and carbon deposits, resulting in clean rCFs.

Inert atmosphere pyrolysis, while effective in minimizing carbon fiber oxidation, leads to reduced resin decomposition and the formation of carbon deposits on the carbon fiber surface, which hinders the reusability of the rCFs. To address this issue, the introduction of a certain amount of oxygen is necessary to enhance resin decomposition and remove the carbon deposits on the fiber surface. López et al. [36] performed pyrolysis of prepreg in an air atmosphere at temperatures ranging from 500 to 700 °C, resulting in a diverse range of gas and liquid compounds as resin degradation products, with the molecular weight of liquid products ranging from 92 to 408 amu. Under optimal conditions, the tensile strength of the rCFs was only 70% of the vCFs, and the surface carbon–oxygen functional groups significantly decreased. The first commercially implemented aerobic pyrolysis recycling system was established by Recycled Carbon Fiber Ltd. in the United Kingdom [58], where the tensile strength of the rCFs was relatively low, retaining approximately 70% of the vCFs’s tensile strength. It was observed that the tensile strength of the rCFs varied significantly depending on their location within the recycling furnace, likely due to uneven heating. Although aerobic pyrolysis increased resin degradation rates, the recycling process led to significant oxidative reactions in the rCFs, resulting in a substantial decline in their mechanical properties. Consequently, the tensile strength of the rCFs obtained through aerobic pyrolysis showed a substantial decrease, reaching only 70–85% of the vCFs’ strength, even under laboratory conditions [37,38,39].

The results indicate that, in gas pyrolysis, resin degradation is not thorough enough and rCFs surfaces tend to accumulate carbon deposits under an inert atmosphere. Oxygen can accelerate resin degradation and prevent carbon deposition, but it leads to significant mechanical losses and considerable variability in the properties of the rCFs. Additionally, the resin products consist of various gases and small molecular compounds. Gas pyrolysis offers relatively acceptable levels of defatting and mechanical properties in the rCFs under these conditions, and it is less restrictive in terms of CFRP waste types, while also boasting a high degree of automation in the recovery equipment. As a result, it is easier to scale up, and currently, it is gradually transitioning from laboratory experimentation to practical applications.

#### 2.2.3. Microwave Pyrolysis Method

Microwave pyrolysis has been developed based on gas-phase pyrolysis. Conventional pyrolysis involves gradual heating from the outer surface to the inner part, resulting in non-uniform heating. In contrast, microwave pyrolysis allows the material to absorb microwave energy and heat uniformly and rapidly. Lester et al. [59] utilized microwave pyrolysis to heat 3 g of pre-impregnated waste particles in a 3 kW microwave reactor for 8 s. A stable nitrogen gas flow of 5 L/min was continuously introduced to prevent the combustion of carbon fibers during the heating process. The rCFs obtained were relatively clean, with a residual resin content of 2.8%. However, compared to the vCFs, the mechanical properties of the rCFs significantly decreased, with reductions of 20% in tensile strength, 12% in tensile modulus, and 33.6% in surface roughness. Hao et al. [60] used microwave pyrolysis on prepreg between 450–650 °C and found residual carbon on the surface of the rCFs. To remove the carbon residues, additional heating at 550 °C in an air atmosphere for 30 min was required. The tensile strength of the rCFs decreased by nearly 20%, while the surface elements and functional groups were similar to those of the vCFs. The main gas products generated during the recycling process were CO, CO_2_, and CH_4_, while the liquid products consisted mainly of phenolic and aromatic compounds. Ren et al. [61] employed microwave heating at 500 °C in an argon atmosphere for 15 min to recover carbon fibers from CFRP. However, the rCFs contained more residual resin and carbon deposits on their surface, necessitating further oxidation at 550 °C for 30 min.

The biggest advantage of microwave pyrolysis lies in its rapid heating rate, enabling the resin to be pyrolyzed and carbon fibers to be recovered in a short period. However, a significant drawback is the substantial accumulation of carbon deposits on the surface of the rCFs, necessitating additional high-temperature oxidation treatment. Moreover, there is a substantial reduction in mechanical properties, with tensile strength typically reaching about 80% of that of the vCFs. Additionally, the amount of carbon fibers that can be recovered in a single process is limited. Currently, there is relatively scarce literature on directly recycling CFRP using this method. Some researchers have shifted their focus and utilized this technique as an auxiliary means for recycling carbon fibers, employing it to heat reaction solutions and facilitate the transfer of these solutions within the resin.

### 2.3. Research on Chemical Solvent Degradation Method

The chemical solvent degradation method involves the synergistic action of chemical solvents and high-temperature heating (sometimes under high-pressure conditions) to degrade the resin within the solvent, enabling the filtration and recovery of carbon fibers. The key to solvent decomposition lies in designing appropriate solvents and reaction conditions to facilitate resin degradation. Chemical solvent decomposition methods mainly include super/subcritical fluid methods, atmospheric solvent methods, and organic base/organic solvent methods, among others.

#### 2.3.1. Super/Subcritical Fluid Method

Under super/subcritical conditions, solutions such as water and alcohols possess both gas-like and liquid-like properties, exhibiting compressibility, flowability, low viscosity, and high diffusivity [62]. The super/subcritical fluid method utilizes such solutions to degrade the resin and recover carbon fibers. The solution can only achieve a super/subcritical state when both temperature and pressure reach their intrinsic critical values. In current research, water or alcohols are commonly selected as super/subcritical fluids. Water has a critical temperature of 373 °C and a critical pressure of 22.06 MPa. On the other hand, the critical temperature of alcohols is approximately 240 °C, and the critical pressure varies within the range of about 5–8 MPa, depending on the specific type of alcohol used.

Hernanz et al. [40] conducted the recovery of prepreg under super/subcritical water conditions at 250–400 °C and 4–27 MPa. However, the degradation efficiency of epoxy resin was unsatisfactory. Under the optimal conditions (400 °C, 27 MPa) with a reaction time of 30 min, the removal rate of epoxy resin was 79.3%. The supercritical recovery device is depicted in Figure 2c. Meng et al. [63,64] studied the recovery of carbon fibers under supercritical water conditions, exploring the influence of various experimental parameters (temperature, waste-to-water ratio, pressure, and reaction time) on the efficiency of epoxy resin degradation. They found that temperature and pressure had the most significant impact on recovery efficiency. The research conducted by the Pickering team [41,42,65] under super/subcritical conditions investigated the decomposition of resins using methanol, ethanol, n-propanol, and acetone and highlighted the role of solvent solubility in determining the reaction rate of the recovery system. Among the solvents tested, n-propanol exhibited the closest Hildebrand parameter to that of epoxy resin, making it the most suitable chemical solvent for recovering CFRP from an epoxy resin matrix. Henry et al. [43] utilized water or water/ethanol as solvents for CFRP recovery under 350–400 °C and 25 MPa conditions, revealing that higher reaction temperatures led to a greater variety of organic compounds in the resin degradation products. Moreover, the oxidation of the rCFs in water was more pronounced, resulting in a significant reduction in surface roughness and an increase in the content of carbon–oxygen functional groups.

Under super/subcritical conditions, using only water or alcohol for CFRP recovery does not yield significant resin degradation. As a result, researchers devise auxiliary conditions, such as introducing gases and adding catalysts, to facilitate resin degradation. Fromonteil et al. [44], under supercritical water conditions at 410 °C and 24 MPa, introduced a certain amount of oxygen to recover CFRP, enhancing resin degradation and ultimately achieving carbon fiber recovery. The resin degradation products included various liquid-phase compounds such as alcohols, aldehydes, and gaseous-phase compounds like alkenes. Sato et al. [66], at 440 °C and 2 MPa, continuously introduced nitrogen gas and used CaCO_3_, Na_2_CO_3_, and Fe_2_O_3_-S as catalysts to degrade polycarbonate resin in tetrahydronaphthalene, trans-decalin, and cyclohexanol solvents. The degradation products consisted of gases, oil-phase products, and liquid-phase products, with the oil-phase products containing some monomers such as phenol and 4-isopropylphenol. Liu et al. [45], under subcritical conditions at 210 °C and 4.8 MPa, used potassium hydroxide as a catalyst to recover anhydride-cured resin in methanol. After 1 h of reaction, the resin degradation rate reached 83.1%, and the degradation products included esters, acids, and a series of benzyl compounds. The use of potassium hydroxide as a catalyst significantly lowered the degradation temperature of the epoxy resin.

The advantages of the super/subcritical fluid method include a remarkably fast resin degradation rate and relatively high resin removal efficiency. However, the disadvantages are also evident. For instance, the high-pressure and high-temperature conditions demand stringent requirements for the quality of recovery equipment, making it challenging to scale up the process and it results in substantial energy consumption. Furthermore, the degradation products of the resin are diverse with low molecular weight, and under super/subcritical conditions, rCFs sustain significant damage.

#### 2.3.2. Atmospheric Pressure Solvent Method

Faced with the challenges of super/subcritical conditions, some researchers have shifted their focus to degrading resins and recovering carbon fibers under atmospheric pressure using the synergistic effects of high temperature with solvents and electric current with solvents. Lee et al. [46] employed a flow system using different concentrations of nitric acid as a solvent to recover carbon fibers from CFRP at temperatures ranging from 80 to 100 °C. The optimal parameters determined from the experiments were a nitric acid concentration of 12 mol/L, a temperature of 90 °C, and a reaction time of 6 h. Under these conditions, the rCFs exhibited minimal surface defects, with a tensile strength of 97% compared to the vCFs. Nie et al. [47] immersed CFRP in molten NaOH at temperatures between 285 and 330 °C while continuously introducing nitrogen gas at a flow rate of 20 mL/min. At 330 °C, the epoxy resin was completely decomposed after 30 min, and the tensile strength loss of the rCFs was minimal, reaching 95% of the vCFs’s strength. However, the weight ratio of NaOH to the composite material was 25, resulting in an excessively large amount of NaOH usage that cannot be recycled, leading to significantly high recovery costs. Although using high concentrations of acids and bases as reaction solvents achieved favorable results, these highly concentrated acids and bases are extremely oxidizing and corrosive. They not only pose significant potential risks to operators but also demand stringent anti-corrosion and anti-leakage requirements for the equipment.

Subsequently, researchers conducted recovery studies using a mixture of catalysts and highly soluble organic solvents [67]. Deng et al. [48] utilized a mixed solution of 1-propanol, water, and acetone as the solvent and KOH as the catalyst to recover carbon fiber-reinforced phenolic resin–boron composites in a high-pressure autoclave at 280 °C. After a continuous reaction for 4 h, the resin degradation rate reached 98.3%. The resin degradation products consisted of liquid and gas products, with esters (47.92%) and amides (32.62%) being the predominant components. The variety of compounds was complex, with the maximum molecular weight being only 238.28 Da. Furthermore, the rCFs had a minimal amount of resin on their surface, and the content of oxygen-containing functional groups decreased. Zhao et al. [49] first subjected CFRP to a 40 min swelling treatment in ice acetic acid at 90 °C, followed by recovery in monoethanolamine containing 10 wt% KOH. After reacting at 160 °C for 90 min, the resin degradation rate reached 98.82%. The rCFs underwent mild oxidation, with a slight increase in surface roughness and an increase in oxygen-containing functional groups, resulting in a slight improvement in wettability but a 6.5% reduction in tensile strength. Yang et al. [50] immersed CFRP in a mixture of polyethylene glycol and NaOH at 200 °C for 4 h, achieving a resin degradation rate of 84.1–93.0%, and the tensile strength of the rCFs reached 96% of the vCFs. It can be observed that the chemical characteristics of the catalysts/organic solvents are correlated with epoxy resin [68]. Therefore, a good resin degradation performance leads to less damage to the rCFs. Moreover, the reaction temperature has generally decreased to below 300 °C, with inorganic base KOH playing a significant role as the catalyst.

**Figure 4:**
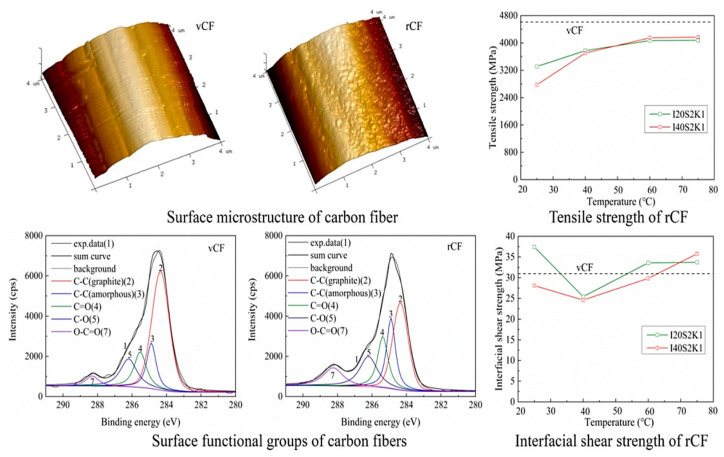
Properties of recycled carbon fibers [3,51].

The Zhu team [3,51,69] pioneered the use of the synergistic effect of current and alkaline solutions for CFRP recovery, as shown in Figure 2d, setting a precedent for electrochemical CFRP recovery. They employed low-concentration NaCl solution and KOH catalyst under a 3V direct current to recover CFRP at temperatures ranging from 25 to 75 °C. The CFRP was completely degraded, and carbon fibers were recovered by the ClO^−^ generated, which broke the C-N bonds in the resin molecular chain. The performance of the rCFs is shown in Figure 4. The surface roughness of the rCFs increased, and the functional group content rose, with tensile strength and interfacial shear strength reaching 89.46% and 121% of the vCFs, respectively. Electrochemical methods can typically be performed at room temperature and atmospheric pressure, without requiring stringent recovery conditions. The loss of rCFs is minimal, maintaining high tensile strength. Moreover, during the recovery process, the rCFs surface undergoes electrochemical modification, leading to a significant enhancement in interfacial shear strength. Hence, electrochemical methods represent gentle, environmentally friendly, and functional recovery techniques, with the potential for large-scale applications. However, when the current–voltage exceeds 5V, both the resin and carbon fibers suffer significant degradation [70]. The Oshima team [71,72], in their attempts to recover CFRP in electrolytes such as NaCl, KCl, NaOH, and KOH using currents ranging from 2.5 to 15 V, found simultaneous degradation of the resin and carbon fibers. The degree of resin degradation was low, making it difficult to separate the carbon fibers from the resin, rendering them no longer usable. Thus, the challenge with the electrochemical method lies in selecting an appropriate current to minimize the degradation impact on rCFs and designing suitable electrolytes and catalysts to enhance resin degradation efficiency.

The atmospheric pressure solvent method does not require high-pressure conditions, and the reaction temperature is typically below 300 °C, making it a relatively mild recovery approach. The tensile strength of the carbon fibers recovered through the atmospheric pressure solvent method is usually above 85%. Additionally, it allows the recovery of resin degradation products with relatively large molecular weights, thus increasing the potential for studying resin degradation mechanisms and enhancing resin recovery possibilities. Furthermore, the atmospheric pressure solvent method has demonstrated the significant catalytic role of alkalis such as KOH in resin degradation, making it an important direction for the development of CFRP recovery technologies.

#### 2.3.3. Organobase/Organosolvent Method

**Figure 5:**
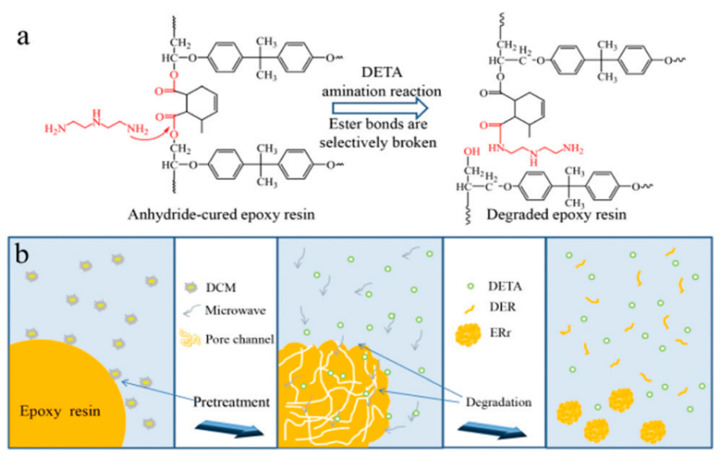
(**a**) Chemical structure and amination process of the resin; (**b**) Pretreatment and microwave-assisted amination process of the resin [55].

In recent years, researchers in the field of resin recovery have explored a new approach by using organic strong bases and highly soluble organic solvents to recover materials such as CFRP [52] and plastics [73]. Zhang et al. [53] investigated the recovery of anhydride-cured epoxy resin by using a mixture of 10 different organic bases with ethylene glycol (EG). Among them, N-methyl-4-piperidinol showed the best resin degradation performance, achieving a degradation rate of 99.7% at 180 °C for 3 h. Kuang et al. [54] recovered anhydride-cured epoxy resin by immersing it in a mixture of 1,5,7-triazabicyclo [4,4,0]dec-5-ene (TBD), EG, and N-methyl-2-pyrrolidone (NMP). The glass transition temperature (*T*_g_) of the resin was 157 °C, and at a reaction temperature of 170 °C, the degradation rate was about 95%, obtaining oligomers as active components for manufacturing new resins. Analysis using Nuclear Magnetic Resonance (NMR) and Fourier Transform Infrared Spectroscopy (FTIR) suggested that the degradation of the resin was due to the cleavage of ester bonds in the molecular structure. NMP could expand the resin, making it easier for TBD and EG to enter the resin network and improve the degradation rate of the resin. Zhao et al. [55] first immersed anhydride-cured epoxy resin particles in dichloromethane for 48 h to create a porous structure and reduce *T*_g_ from 134.7 °C to 118.05 °C. Then, the treated resin was placed in diethylenetriamine (DETA) at 130 °C and heated under microwave irradiation at 300 watts for 50 min for degradation, achieving a degradation rate of 99%. Analysis using NMR and FTIR revealed that the degraded resin contained amide and hydroxyl groups after ester bond cleavage, as shown in Figure 5. The average molecular weight of the degradation product was 1226 Da, and *T*_g_ was 52.05 °C. It was mixed with unreacted DETA to manufacture new resins as curing agents. DETA acted as both a catalyst and a reactant, while microwave heating not only rapidly heated the reaction mixture and resin particles but also enhanced the penetration of the reaction solution. Additionally, monoisopropanolamine was used to recover double maleimide-based resins with a *T*_g_ of approximately 98 °C at 160 °C [52]. The average molecular weight of the degradation product was 4181 Da and was utilized to produce new resins. Based on the NMR and FTIR analysis of the resin degradation products, it was inferred that the cleavage of C-N and C-C bonds led to resin degradation.

The above-mentioned mixture of organic bases and organic solvents can typically expand the resin, reducing the difficulty of the reaction solution entering the interior of the resin and enhancing the transfer efficiency of the reaction solution. EG as a carrier for the strong base further improves the resin degradation efficiency, allowing the reaction temperature to be reduced to below 200 °C. The organic base/organic solvent recovery method mainly produces oligomers as resin degradation products with molecular weights typically ranging from 400 to 4181 Da, which can be directly reused in the manufacturing of new resins, signifying a significant advancement in recovery technology. Additionally, obtaining the powdered solid of resin degradation products provides a relatively accurate material basis for studying the resin degradation mechanism. By using Nuclear Magnetic Resonance (NMR) and Fourier Transform Infrared Spectroscopy techniques, the molecular structure of resin degradation product fragments and the changes in functional groups before and after resin degradation can be analyzed to infer the resin degradation mechanism. However, the reaction temperature for the organic base/organic solvent method still needs to be higher than the resin’s glass transition temperature (*T*_g_) for effective resin degradation. Moreover, this recovery method is specific to a single type of resin, and its effectiveness for other types of resin remains unverified. Furthermore, organic bases/organic solvents not only have significant impacts on human health and the environment but are also expensive, making it difficult to widely apply them in recovering CFRP. Compared to other recovery techniques, the organic base/organic solvent recovery method offers milder recovery conditions, extremely high resin degradation efficiency, larger molecular weight resin degradation products, and an in-depth study of resin degradation mechanisms, making it worthwhile for researchers to learn from and further improve.

## 3. Research and Analysis of Recycled Carbon Fiber Reuse

The research on the reutilization of rCFs began relatively late, starting in the first decade of the 21st century. Researchers in the materials-related field collected prepreg or discarded CFRP particles from the production process to use as reinforcing phases in the manufacturing of composites, but these composites often exhibited poor mechanical properties. With the development of recycling techniques, researchers gained access to rCFs obtained through pyrolysis or solution degradation methods. These rCFs had low resin content and strong adhesion to the resin, resulting in significantly improved mechanical properties of CFRP produced using them. Currently, using rCFs to manufacture resin-based composites remains mainstream, while some researchers have begun exploring the application of rCFs in the production of carbon fiber felts. In recent years, researchers in the civil engineering field have also started using rCFs to reinforce cementitious composites, studying the mechanical properties, electrical conductivity, and environmental impact of the composite materials. The rCFs primarily come from collected waste carbon fibers and mechanically recycled CFRP particles. This paper provides an overview of the reutilization of rCFs in the field of fiber composites and cementitious composites.

### 3.1. Research on the Reuse of rCFs in Fiber Composites

**Figure 6:**
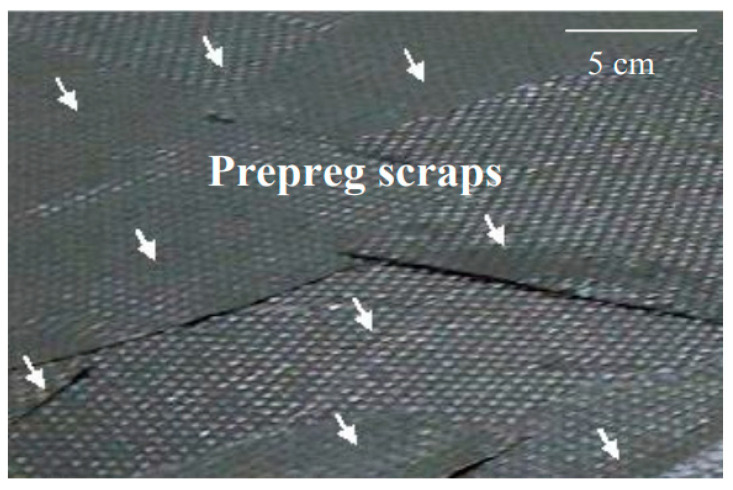
Laminate made from waste prepreg [75].

Researchers have utilized collected carbon fiber waste, prepreg scraps, or rCFs to re-manufacture CFRP. The mechanical properties, such as tensile strength, flexural strength, and impact strength, of the re-manufactured CFRP have been investigated to explore the utilization of rCFs. Regarding the manufacturing of CFRP using waste carbon fibers and prepreg scraps, Aravindan et al. [74] employed high-performance discontinuous fiber technology to re-manufacture waste fiber bundles into highly aligned discontinuous fiber prepreg tapes, which were then used to produce unidirectional laminates. Compared to virgin carbon fiber reinforced plastics (vCFRP), the recycled carbon fiber reinforced plastics (rCFRP) showed reductions in tensile strength, stiffness, and failure strain by 85.9%, 72.4%, and 47.4%, respectively. Souza et al. [75] used uncured prepreg to produce different sizes and shapes of laminates, as shown in Figure 6. They found that the tensile strength, flexural strength, and compressive strength of rCFRP decreased by 13%, 56%, and 23% compared to vCFRP. Mohama et al. [56] utilized waste prepreg particle-reinforced polypropylene, where fully cured prepreg was first crushed into 5–10 mm fine particles, and then composite materials were prepared using 3–5% by mass of these particles. However, the adhesion between prepreg and polypropylene was poor, resulting in composite materials with tensile strength equivalent to pure PP. As seen from the results, due to discontinuity, waste carbon fibers, and CFRP often struggle to fully harness the mechanical advantages of carbon fibers when manufacturing composites. Additionally, the poor bonding between the cured resin on the surface of waste carbon fibers/CFRP and the new resin further contributes to the significant reduction in the mechanical properties of rCFRP.

**Figure 7:**
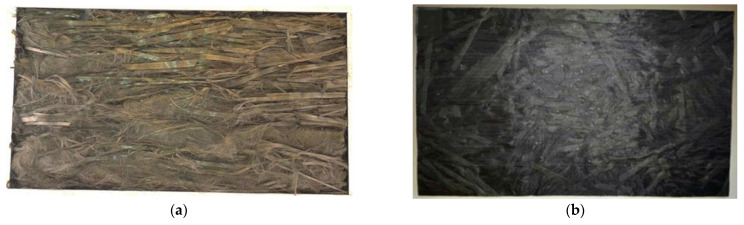
(**a**) Recycled carbon fibers bundle before brushing resin; (**b**) rCFRP board [76].

Regarding the preparation of CFRP using rCFs, Schwarz et al. [34] conducted a 2 h pyrolysis of prepreg in a nitrogen atmosphere at 480–560 °C, resulting in a resin content of 22.6% in the rCFs at 560 °C. SEM analysis revealed an abundance of resin particles on the surface of the rCFs. The tensile strength and elastic modulus of rCFRP were both below 50% of vCFRP. Giorgini et al. [35] treated prepreg and CFRP waste in a nitrogen atmosphere at temperatures ranging from 450 to 600 °C but achieved poor resin removal. Short rCFs and vCFs were used to manufacture new composite materials, but the resin failed to fully impregnate the rCFs. Tensile tests indicated delamination between the rCFs and the resin, with rCFRP exhibiting fracture stress, Young’s modulus, and elongation at fracture of 33.3–81.6%, 68.4–94.7%, and 41.1–91.6% of vCFRP, respectively. Oliveux et al. [76] used solvent-recovered carbon fiber bundles and vCFs bundles to prepare carbon fiber sheets, as shown in Figure 7. Residue on the surface of the rCFs hindered the bonding between the carbon fibers and the resin, and the low alignment of the rCFs resulted in voids and resin-rich regions. The tensile strength and modulus of rCFRP were approximately 63.1–72.4% and 85–137.5% of vCFRP, respectively.

Regardless of pyrolysis recycling or solvent degradation, if the surface of the rCFs still contains a significant amount of resin, it will affect the bonding between the rCFs and the resin, ultimately leading to a decrease in mechanical properties such as tensile strength and flexural strength, as confirmed by previous studies [77,78]. When the resin on the surface of the rCFs is completely removed, and the surface becomes hydrophilic, it can enhance the bonding between the rCFs and the resin. Deng et al. [48] obtained rCFs with very little resin on the surface and increased contact angle under subcritical conditions. During the manufacture of CFRP, they found that the vCFs and the resin interface tend to form resin-rich regions and voids, resulting in stress concentration. Due to the good wetting properties between the rCFs and the resin, a stronger interface was formed, leading to an increase of 102.4% in the flexural strength of rCFRP compared to vCFRP. Furthermore, the dispersion of rCFs can also have adverse effects during the production of CFRP. Guo et al. [79,80] prepared CFRP using 10 mm long rCFs and vCFs separately and found that the clustered structure of rCF resulted in lower dispersion compared to vCF, leading to brittle fracture behavior in rCFRP and a decrease of 88.14% in flexural strength compared to vCFRP.

**Figure 8:**
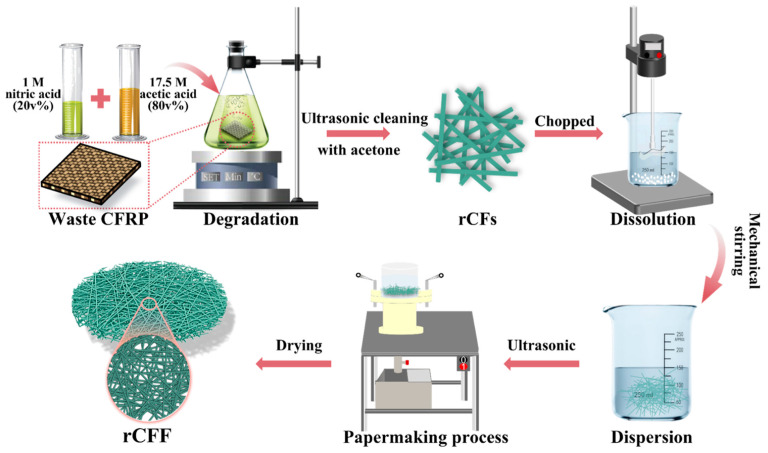
Schematic illustration for the fabrication procedure of recycled carbon fiber felt [81].

Additionally, the utilization of rCFs has been extended in recent research. Hu et al. [81] further ultrasonically dispersed the resin-free carbon fibers obtained from solvent recycling and then utilized a papermaking method to produce recycled carbon fiber felts decorated with cationic polyacrylamide (rCFF/CPAM), as shown in Figure 8. The rCFs exhibit conductivity and good dispersibility, resulting in rCFF/CPAM demonstrating significant conductivity of 140.06 S/m, highly efficient EMI shielding effectiveness of 66.15 dB, and a special SEA/SET ratio of 83.8% at 0.875 mm. Moreover, the recycled carbon fiber felt possesses advantages such as lightweight, flexibility, environmental friendliness, and cost-effectiveness.

In conclusion, in the field of fiber-reinforced composites, researchers have utilized rCFs for manufacturing CFRP and carbon fiber felts. Due to the discontinuity of the rCFs and their high resin content, rCFRP exhibits a significant decrease in mechanical properties such as tensile strength, flexural strength, and impact strength. In contrast, the recycled carbon fiber felt demonstrates excellent conductivity and efficient shielding effectiveness. The application of rCFs in the fiber-reinforced composites field is a promising utilization method that allows for the “rebirth” of discarded CFRP, enabling it to continue fulfilling its intended function.

### 3.2. Research on the Reuse of rCFs in Cementitious Composites

In recent years, rCFs have been utilized by some researchers in the field of cementitious composites. They have been employed to enhance the mechanical properties and conductivity of cementitious composites. Additionally, an assessment of the environmental impact of incorporating rCFs in cementitious composites has been conducted.

#### 3.2.1. Research on Carbon Fiber Reinforced Cementitious (CFRC) Composites

Cementitious materials, composed of cement, aggregates, mineral admixtures, and water [82], possess advantages such as strong adhesion, good plasticity, high compressive strength, and excellent durability, making them fundamental materials in civil engineering. However, as brittle materials, cement-based materials have low flexural and tensile properties, making them prone to cracking. Moreover, conventional cementitious composites exhibit a resistivity range of 104 to 107 Ω m, placing them between insulators and semi-conductors, with nearly no conductivity under fully dried conditions [83]. By incorporating high-strength carbon fibers, which possess excellent thermal and electrical conductivity, into cementitious materials, it is possible to enhance their compressive [84,85,86], flexural [87,88,89], tensile [90,91], and crack resistance [92,93], as well as their fatigue properties [94,95]. Additionally, carbon fibers act as conductive elements, significantly reducing the resistivity of the composites. This expansion of conductivity [96,97], thermal conduction [98,99], sensor [100,101], and electromagnetic shielding [102,103] attributes in cementitious composites open up new possibilities for various functional applications.

However, the vCFs are inert materials, with a hydrophobic surface and low density, making it difficult to disperse them within the cement matrix [104,105]. Additionally, the weak chemical reactivity and smooth surface of the vCFs result in poor adhesion with cement hydration products, thereby reducing the interfacial bond strength with the cement matrix [106,107,108]. Consequently, carbon fibers tend to agglomerate in the cement matrix, leading to reduced flowability and workability of CFRC composites [97,109]. Moreover, the agglomeration introduces more air voids, causing the formation of pores in CFRC [110] and further weakening the bond between carbon fibers and the cement matrix. As a result, the enhancement of mechanical and electrical properties of CFRC is diminished [111,112,113], especially compressive strength [114,115,116]. This is because the clustering of carbon fibers results in the generation of numerous cracks and voids in the nearby matrix [104,117], and insufficient contact between clustered carbon fibers and the matrix leads to the failure of load transfer between them, impeding the crack-inhibiting effect of carbon fibers. In contrast, rCFs are often subjected to oxidation, resulting in a hydrophilic surface with abundant oxygen-containing functional groups, effectively improving their dispersion in the cement matrix [118]. Moreover, the wider and deeper grooves on the surface of rCFs provide effective nucleation sites for nearby hydration products, enhancing the mechanical anchoring effect between rCFs and the cement matrix [118,119].

Additionally, while carbon fibers can significantly enhance the mechanical and electrical properties of cementitious composites, their usage requires consideration of cost issues. Taking the application of carbon fibers in concrete as an example, the price of carbon fibers is approximately USD 33/kg [6,28], while the cost of C40 concrete in the Shenzhen area is about 700 CNY/m^3^. A total of 1 m^3^ of concrete contains approximately 500 kg of cement, and if the carbon fiber dosage is calculated as 1% of the cement mass, 1 m^3^ of concrete would require 5 kg of carbon fibers. The cost of carbon fibers would be approximately 1155 CNY, which already far exceeds the cost of the concrete itself. This significantly increases the cost of carbon fiber-reinforced concrete, hindering the application of carbon fibers in the field of civil engineering.

#### 3.2.2. Research on Recycled Carbon Fiber Reinforced Cementitious (rCFRC) Composites

Faced with the drawbacks of vCFs, such as low surface chemical activity, hydrophobicity, and smoothness, leading to difficulties in dispersion within the cementitious matrix and weak bonding with cement, some researchers have attempted to utilize rCFs to reinforce cementitious composites. Currently, researchers have utilized collected CFRP waste or mechanical rCFs, further crushing them before adding them to cementitious composites. The focus of the research has mainly been on the mechanical properties of rCFRC composites, with only a limited amount of attention given to the composites’ electrical conductivity and environmental impact.

1. Research on mechanical properties

**Figure 9:**
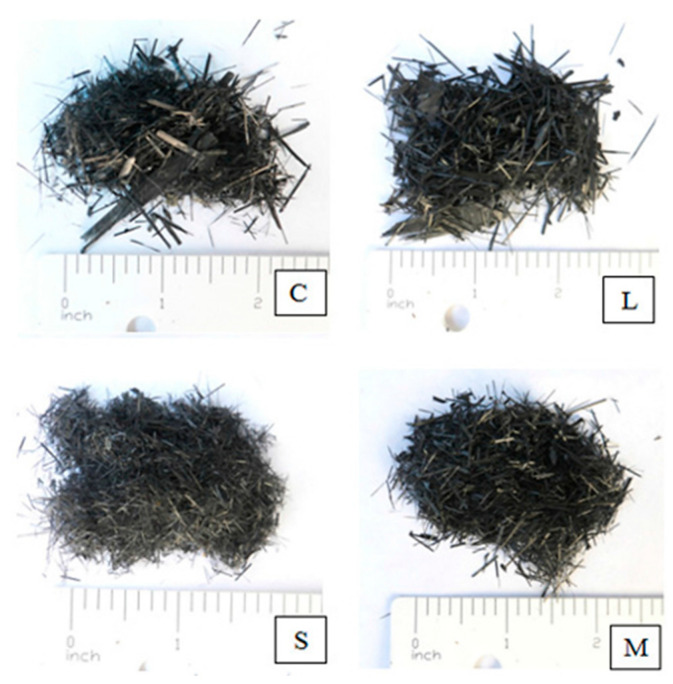
Four different sizes of CFRP were included in the experiment: C-combined, L-large, S-small, and M-medium fraction [120].

Currently, research on the mechanical properties of CFRC mainly includes compressive strength, flexural strength, and tensile splitting strength. Rangelov et al. [120] employed CFRP particles to enhance the performance of previous concrete. The CFRP particles were classified into four distinct size categories: particles smaller than 3.35 mm (referred to as “combined”), particles smaller than 3.35 mm but larger than 2 mm (referred to as “large”), particles smaller than 2 mm but larger than 0.841 mm (referred to as “medium”), and particles smaller than 0.841 mm (referred to as “small”), as illustrated in Figure 9. It was found that the porosity of the composite material could be reduced, leading to an increase in permeability. Additionally, the compressive strength, tensile splitting strength, and elastic modulus increased by 4–11%, 11–46%, and 6–45%, respectively. Xiong et al. [121] investigated the influence of 0–1.5% CFRP sheets and rubber on the mechanical properties of concrete. They found that CFRP sheets reduced the slump of concrete but slightly increased the compressive strength (up to 5.5%) and flexural strength (up to 10%). Moreover, they significantly improved ductility, flexural toughness, and impact resistance. Mastali et al. [122] used 10–30 mm length and 0.5–2% content of CFRP sheets to reinforce self-compacting concrete, resulting in a 50% increase in maximum compressive strength and a 60% increase in maximum flexural strength. However, the slump decreased by 15%, significantly reducing the workability of the concrete. SEM images indicated that the failure of the recycled CFRP sheets occurred mainly due to debonding. The reduced workability of the composite material was primarily attributed to the non-continuous state of the added CFRP waste, and its surface lacked chemical activity, preventing a strong bond with the cementitious slurry, thus impeding the flowability of the composite slurry.

**Figure 10:**
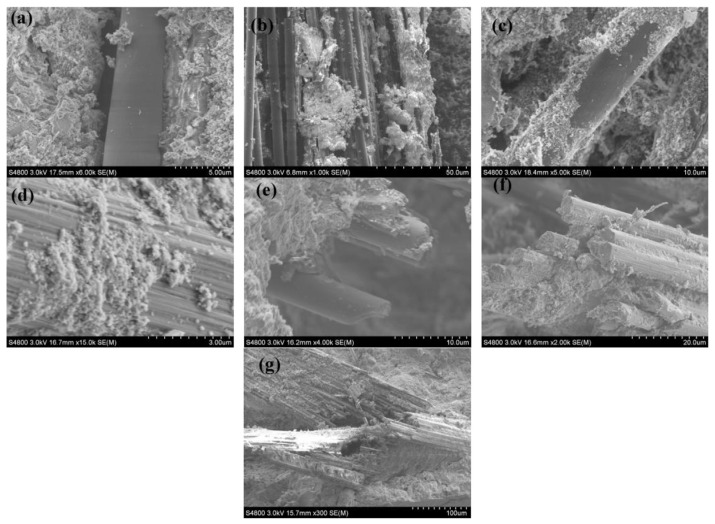
Morphology of rCF with different treatments: (**a**) untreated, (**b**–**d**) treated by 1 mol/L NaOH solution, (**e**,**f**) treated by 2 mol/L NaOH solution, and (**g**) treated by 3 mol/L NaOH solution [118].

The epoxy resin residue on the surface of CFRP waste is an organic substance that can weaken the interfacial bond between carbon fibers and the cementitious matrix, thus adversely affecting the mechanical properties of CFRC [123,124,125,126]. Therefore, researchers have attempted to remove the resin from the surface of CFRP waste. Wang et al. [118] treated waste CFRP particles with a NaOH solution and used them to reinforce cement mortar. They found that a 1 mol/L NaOH solution could partially remove the epoxy resin residue on the surface of rCFs, making the surface of the waste carbon fiber particles rougher and allowing for better bonding with hydration products, as shown in Figure 10. Compared to untreated CFRP particles, the compressive strength of cement mortar increased by approximately 6%. This indicates that removing the cured resin from the surface of CFRP waste is an effective method to enhance its bond with the cementitious matrix. Li et al. [127] removed the remaining resin and carbonaceous impurities on the surface of rCFs obtained through thermal decomposition using an electrochemical anodic oxidation method. This process enhanced the bond between rCFs and the fly ash-activated composite matrix, resulting in a 185% increase in single-fiber interfacial shear strength and a 25% and 19% increase in compressive and flexural strength of the fly ash-activated composite, respectively. Although waste carbon fibers’ surfaces do not contain cured resin, surface sizing agents still negatively influence the interfacial bond strength [128]. Therefore, rCFs can effectively improve the macroscopic mechanical properties of cementitious composites, and rCFs without sizing agents or resin impurities on their surface exhibit better-reinforcing effects.

2. Research on electrical conductivity

Some researchers have investigated the influence of waste carbon fibers on the electrical conductivity of cementitious composites. Faneca et al. [129] reinforced high-strength concrete with waste carbon fiber bundles and CFRP sheets, and the results indicated that both carbon fibers and CFRP sheets reduced the workability of concrete and introduced more porosity. When the carbon fiber bundle content was in the range of 0.2% to 0.8%, the resistivity values ranged from 3 Ω m to 0.6 Ω m, and the electrical conductivity showed no significant difference compared to carbon fiber-reinforced concrete reported in the other literature. Overall, the bundle-shaped carbon fibers exhibited slightly better enhancement of concrete’s electrical conductivity than CFRP sheets. Belli et al. [130] used waste carbon fibers to enhance cement mortar, and the results showed that when the carbon fiber content was in the range of 0.1% to 0.2%, the resistivity of vCFRC decreased from 5491 Ω m to 2070 Ω m. Under the same conditions, the resistivity of rCFRC decreased from 1392 Ω m to 355 Ω m, indicating that waste carbon fibers were more effective in enhancing the mortar’s electrical conductivity compared to vCFs. Therefore, rCFs not only significantly improve the electrical conductivity of cementitious composites but also outperform vCFs in this regard.

3. Research on environmental impact

Furthermore, the environmental impact of using waste carbon fiber to reinforce cementitious composites has been investigated by researchers through lifecycle assessment studies. It was found that using CFRP sheets to enhance concrete can effectively reduce CO_2_ emissions [121]. Vitale et al. [131] utilized prepreg waste material to reinforce cementitious materials and observed not only an improvement in the mechanical performance of the composite but also reductions of approximately 12%, 11%, and 11% in carbon emissions, fossil energy consumption, and inorganic respiratory emissions, respectively. Akbar et al. [132], through a lifecycle assessment, suggested that by incorporating 1% rCFs while substituting 10% of cement with silica fume in cementitious composites, the overall global warming potential index for CO_2_ emissions decreased by 13.69% compared to ordinary cementitious mortars. Additionally, replacing vCFs with rCFs resulted in energy and cost savings of 22% and 70%, respectively. Therefore, the use of rCFs to reinforce cementitious composites can reduce their environmental impact and lead to cost savings.

As reviewed, in the field of cementitious composites, researchers have utilized rCFs to reinforce cementitious composites and investigated the influence of different dosages and lengths on the mechanical and electrical properties of CFRC. The incorporation of rCFs decreases the workability of CFRC but effectively enhances its compressive, flexural, and tensile strengths, as well as its electrical conductivity. Overall, rCFRC slightly outperforms vCFRC, demonstrating the feasibility of using rCFs as a substitute for vCFs. Moreover, rCFs exhibit significant advantages over vCFs in terms of carbon emissions and other environmental impacts, energy consumption, and cost.

## 4. Conclusions and Prospects

Recycling and reusing CFRP waste are critical for the sustainable development of various industries, such as aerospace and civil engineering, as it has caused severe environmental issues. Over the past three decades, recycling CFRP waste has evolved into three main technological systems: mechanical recycling, thermal decomposition recycling, and chemical solvent degradation recycling. These methods effectively separate carbon fibers from the resins, mitigating environmental pollution. However, challenges remain in the recovery of intact carbon fibers and the recycling and utilization of resin degradation products. Currently, the reuse of rCFs is in its early stages, and more in-depth research is needed. The rCFs used often have higher resin content, weaker hydrophilicity on the surface, smaller dimensions, and a scattered morphology. Furthermore, the focus of research mainly concentrates on the macroscopic mechanical properties, with limited exploration of the types of rCFs reuse. Therefore, apart from advancing research in both recycling techniques and rCFs reuse individually, there is a need to strengthen collaborative research between these two areas. By exploring a closed-loop cycle for CFRP waste recycling and rCFs reuse, the fundamental problem of CFRP waste can be effectively addressed. In the field of fiber composites, there is a demand for large-scale resin-free rCFs to produce integrated high-performance CFRP products and enhance their application value. Similarly, the civil engineering domain requires functional and cost-effective rCFs. Research should focus on improving the mechanical properties of rCFs through recycling techniques while imparting more functionality to them. Simultaneously, investigating the reinforced mechanisms of rCFs in fiber composites and cementitious composites will provide valuable feedback to the recycling techniques. Ultimately, the joint development of recycling CFRP waste and reusing rCFs will contribute to solving the CFRP waste problem. Therefore, the following aspects warrant particular attention in future research:

(1) The efficiency of heat and solvent transfer is hindered by the dense structure of the resin, resulting in low resin degradation efficiency. Suitable auxiliary conditions can be investigated to facilitate rapid heat and solvent transfer into the resin. For instance, pre-treatment techniques such as microwave or resin expansion to enhance porosity, as well as highly penetrative reactive solvents, can be explored.

(2) Mechanism of performance evolution in rCFs. Previous studies have indicated that high temperatures, high pressures, corrosive chemical solvents, and electric currents can lead to the deterioration of rCFs performance. However, the mechanisms underlying the deterioration of rCFs remain unclear. Investigating the performance evolution mechanism of rCFs and using it to guide the design of recycling techniques can contribute to reducing or even avoiding the deterioration of rCFs’ performance.

(3) Recovery and degradation mechanism of resin degradation products. At present, the majority of resin degradation products are by-products of rCFs, mainly consisting of various gases and small-molecule compounds, which have virtually no commercial value and require disposal as waste. Moreover, due to the complexity of small-molecule compounds or secondary products in resin degradation, the accurate analysis of resin cleavage sites becomes challenging, making it difficult to research the degradation mechanism of resins. High-molecular-weight resin degradation products, on the other hand, retain the main molecular backbone, enabling a more precise analysis of the resin degradation mechanism. Therefore, a comprehensive approach that considers both carbon fibers and resin recovery is needed. By focusing on the properties of the resin, the corresponding solvents can be designed, and the reaction temperature can be reduced to facilitate the degradation of the resin into high-molecular-weight oligomers, thereby laying the foundation for the recycling and degradation mechanism research of the resin.

(4) Carbon fiber lap splicing and alignment techniques. The small and disordered dimensions of rCFs result in reduced size and increased porosity of rCFRP, leading to diminished mechanical performance and limited practical value. Elongating and aligning the overlapping of rCFs can be beneficial in addressing these issues.

(5) Interface bonding mechanism between rCFs and cementitious matrix. Macroscopic mechanical tests such as flexural strength and tensile strength demonstrate the strong bonding performance of rCFs. However, the underlying mechanism responsible for the improvement in bonding performance remains unclear and requires quantitative analysis at the microscopic level of individual carbon fiber filaments.

(6) Expanding the utilization types of rCFs to enhance the functionality of composites. Current research has predominantly focused on mechanical and electrical properties. However, there is potential to explore functional composites, such as carbon fiber felts with electromagnetic shielding capabilities or thermal insulation boards with heat conduction properties.

In addition to CFRP, hybrid fiber composites, such as combinations involving carbon and glass fibers or carbon and aramid fibers, have wielded significant influence in industries such as automotive manufacturing, owing to their remarkable mechanical properties and cost-effectiveness. As their application continues to expand, the issue of waste accumulation is gradually emerging. Research into the recycling and reutilization of CFRP waste can serve as a guiding pathway for the recovery and reuse of hybrid fibers and other composite waste.

## Figures and Tables

**Table 1:** The key technical indicators of the different recycling methods.

	Recycling Method	Mechanical Recycling Method [8,29,30]	Thermal Decomposition Recycling Method		Chemical Solvent Degradation Method			
		Crush	Fluidized Bed [31,32,33]	Pyrolysis [34,35,36,37,38,39]	Super/Subcritical [40,41,42,43,44,45]	Atmospheric Pressure Solvent [46,47,48,49,50]	Electrochemical [3,51]	Organobase/Organosolvent [52,53,54,55]
Recycling conditions	Temperature (°C)	Room temperature	450–500	400–700	250–450	90–350	23–75	130–200
	Pressure (MPa)	Atmospheric pressure	0.1–0.25	Atmospheric pressure	5–35	Atmospheric pressure	Atmospheric pressure	Atmospheric pressure
	Toxicity	None	None	None	Yes	Yes	None	Yes
Recycling effect	Tensile strength (%)	50–65	50–75	50–85	85–98	85–98	−90	/
	Interfacial shear strength (%)	/	−80	/	88.6–99	/	−120	/
	Degradation rate (%)	/	/	−92.4	79.3–98.6	90–99	99–99.9	95–99.7
	rCFs size (mm)	<10	10–50	−500	10–50	10–50	−200	/
	Resin products	Resin dust	Carbon–oxygen and carbon–hydrogen gases	Carbon–oxygen and carbon–hydrogen gases	Small molecule compounds	Small molecule compounds	Small molecule compounds	Oligomers
Environmental impact	Environmental impact	Dust	CO_2_, dust, and heat	CO_2_, flotsam, and heat	Solvents such as alcohols, acids and bases, and heat	Solvents such as alcohols, acids, and bases	Trace amounts of Cl_2_ and H_2_	Organic bases and organic solvents

Note: “/” means that the item has not been reported in the literature.

## Data Availability

Not applicable.
